# Multifunctional Enkephalin Analogs with a New Biological Profile: MOR/DOR Agonism and KOR Antagonism

**DOI:** 10.3390/biomedicines9060625

**Published:** 2021-05-31

**Authors:** Yeon Sun Lee, Michael Remesic, Cyf Ramos-Colon, Zhijun Wu, Justin LaVigne, Gabriella Molnar, Dagmara Tymecka, Aleksandra Misicka, John M. Streicher, Victor J. Hruby, Frank Porreca

**Affiliations:** 1 Department of Pharmacology, University of Arizona, Tucson, AZ 85724, USA; lavigne@email.arizona.edu (J.L.); gmolnar@email.arizona.edu (G.M.); jstreicher@email.arizona.edu (J.M.S.); frankp@email.arizona.edu (F.P.); 2 Department of Chemistry and Biochemistry, University of Arizona, Tucson, AZ 85721, USA; remesic@email.arizona.edu (M.R.); cyf.ramos@gyrosproteintech.com (C.R.-C.); hruby@email.arizona.edu (V.J.H.); 3 ABC Resource, Plainsboro, NJ 08536, USA; abc_resource@yahoo.com; 4 Faculty of Chemistry, University of Warsaw, Pasteura, PL-02-093 Warsaw, Poland; dulok@chem.uw.edu.pl (D.T.); misicka@chem.uw.edu.pl (A.M.)

**Keywords:** multifunctional activity, peptidomimetics, opioid receptors, kappa receptor antagonists, analgesic effects, plasma stability, template-based alignment modeling

## Abstract

In our previous studies, we developed a series of mixed MOR/DOR agonists that are enkephalin-like tetrapeptide analogs with an N-phenyl-N-piperidin-4-ylpropionamide (Ppp) moiety at the C-terminus. Further SAR study on the analogs, initiated by the findings from off-target screening, resulted in the discovery of **LYS744** (**6**, Dmt-DNle-Gly-Phe(*p*-Cl)-Ppp), a multifunctional ligand with MOR/DOR agonist and KOR antagonist activity (GTPγS assay: IC_50_ = 52 nM, I_max_ = 122% cf. IC_50_ = 59 nM, I_max_ = 100% for naloxone) with nanomolar range of binding affinity (*K*_i_ = 1.3 nM cf. *K*_i_ = 2.4 nM for salvinorin A). Based on its unique biological profile, **6** is considered to possess high therapeutic potential for the treatment of chronic pain by modulating pathological KOR activation while retaining analgesic efficacy attributed to its MOR/DOR agonist activity.

## 1. Introduction

Chronic pain syndromes remain poorly managed due to the inadequate efficacy of currently available drugs or undesirable side effects associated with doses or long-term administration needed to obtain pain relief. There remains a critical need for novel therapies to enhance the efficacy and the safety of treatments for chronic pain. Multifunctional opioid ligands that interact with multiple opioid receptors have demonstrated their therapeutic potential to reduce adverse effects by allowing for the activation of specific receptor conformations and/or signaling pathways promoted as a result of receptor oligomerization or crosstalk [1]. It has been shown that opioid receptors do not act in isolation in vitro or in vivo, and the simultaneous modulation of multiple targets may generate a more desirable drug profile [2,3,4,5,6].

It is now well established that chronic pain induces up-regulation of dynorphin interaction with the kappa opioid receptor (KOR). The physiological actions of the KOR at supraspinal sites include diminished hedonic tone and dysphoria, effects that oppose the positive hedonic actions of mu opioid receptor (MOR) agonists [7,8]. KOR antagonists are likely to enhance MOR-induced analgesia while diminishing MOR-induced liabilities including addictive liability resulting from negative reinforcement [8,9,10]. Based on these findings, KOR antagonists have become a major pharmacological target for the rapid development of therapeutics in response to the opioid crisis, as listed in the NIDA’s Division of Therapeutics and Medical Consequences (DTMC) ten most wanted pharmacological targets [11]. Clinically, buprenorphine, an MOR partial agonist/DOR and KOR antagonist, is used to treat moderate chronic pain and opioid addiction. Its KOR antagonist activity is thought to mediate anti-depressive, stress relieving, and anxiolytic effects, while also possessing reduced abuse potential [12,13].

**Figure 1:**
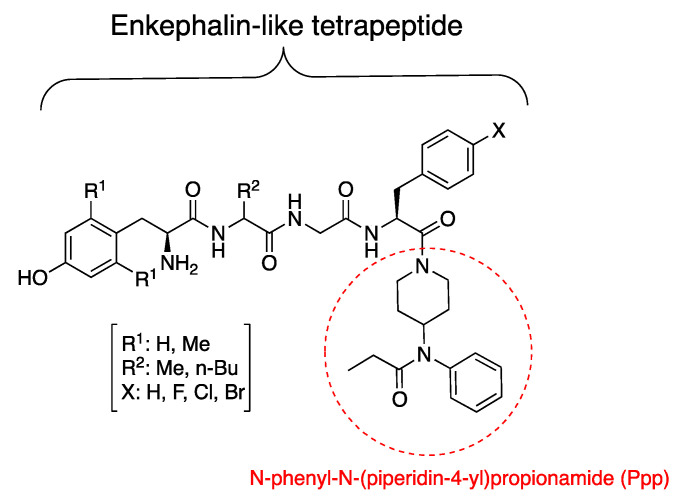
Structures of enkephalin-like tetrapeptide analogs.

In our previous studies, we developed a new series of mixed MOR/DOR agonists with optimized physicochemical properties using an enkephalin-like tetrapeptide scaffold (Figure 1) [14,15]. Despite their therapeutic potential, opioid peptides are limited as clinically viable drugs because of their poor bioavailability, mainly due to poor metabolic stability and low lipophilicity, which limit their ability to cross the blood–brain barrier (BBB). Likewise, enkephalins are highly susceptible to peptide degradation and largely unable to cross the BBB [16]. To enhance the therapeutic potential of peptides as a central nervous system (CNS) drug, it is crucial to improve their metabolic stability and BBB permeability. A recent study demonstrated that fluorinated ENK analogs simultaneously improved druglike physicochemical property and systemic CNS distribution [17]. Likewise, mixed MOR/DOR agonists were designed to overcome the drawbacks of enkephalin-like tetrapeptide (H-Tyr-Gly-Gly-Phe-OH) by incorporating a lipophilic moiety of fentanyl, a N-phenyl-N-(piperidin-4-yl)propionamide (Ppp) group, to the C-terminus [14,15]. This simple modification enhanced the lipophilicities of all analogs (3 < aLogP < 5) together with halogenation of the phenyl ring at position 4. Although lipophilicity is not the only key factor in BBB permeability, it is likely that increasing lipophilicity boosts ligand potential to cross the BBB [18,19,20].

Recently, we discovered that most of the mixed MOR/DOR agonists we previously synthesized interact with KOR as well, which is unusual considering the well-known structure–activity relationships (SAR) of opioid peptides, especially enkephalin and dynorphin analogs. Overall, the substitution of DNle at position 2 and the halogenation of the aromatic ring of Phe at position 4 increased biological activities for the KOR as well as MOR and DOR. Among those analogs, **LYS739 (5)** and **LYS744** (**6**) were the most potent multifunctional ligands, with a partial agonism and a pure antagonism at the KOR, respectively. Considering the negative effects of KOR activation in pain states, analog **6**, showing the new biological profile, may improve the undesirable adverse effects from either pure MOR or KOR agonists [7,8,10]. The analog may offer enhanced efficacy through synergy between the MOR and DOR [1]. Our previous studies demonstrated that **LYS707** (**8**) exhibited potent agonist activities at MOR and DOR (IC_50_ = 0.70 and 2.6 nM in MVD and GPI assays, respectively), and was a ligand with strong anti-hyperalgesic and anti-allodynic effects in SNL rats [14]. To test whether the modifications aided metabolic stability, the lead ligands were first tested for their plasma stability. For a better understanding of the SAR results and the unique KOR antagonism, modeling experiments using template-based alignment modeling (TAM), our new molecular modeling approach, developed recently for the structural correlations and SAR analysis of ligands, was performed on **6** [21].

## 2. Materials and Methods

### 2.1. Synthesis

Enkephalin analogs were synthesized by liquid phase synthesis using N^α^-Boc-chemistry, and their structures were validated by NMR spectroscopy and high-resolution mass spectrometry (HRMS) as described in our previous publications [14,22].

### 2.2. Radioligand Labeled Binding Assays at the KOR

Binding affinities were determined by radioligand competition analysis using [^3^H]U69,593 (1.5 nM) in the transfected HN9.10 cells that express the KOR as previously described [23,24]. The crude cell membranes were diluted to 50 μg of protein per sample in Tris-HCl buffer solution (50 mM, pH 7.4). Radioactivity data were analyzed by nonlinear least-squares analysis using GraphPad Prism. Logarithmic values were determined from nonlinear regression analysis of data collected from at least three independent experiments.

### 2.3. [^35^S]GTPγS Assays

Assays were performed using frozen CHO-KOR pellets, 40 μM GDP, and 0.1 nM [^35^S]GTPγS in Tris buffer as previously described in detail [25]. Data were presented as the mean ± SEM of the percentage of U50,488 stimulation. Values were normalized to the maximum stimulation caused by U50,488. Concentration–response curves were fitted using a nonlinear three-parameter regression curve, and efficacy and/or potency values were reported as mean ± SEM from n = 3 independent experiments for both modes using GraphPad Prism. Agonist: concentration curves of drug incubated were performed as described above. Antagonist: concentration curves of antagonist incubated with protein for 5 min followed by 100 nM of U50,488 and [^35^S]GTPγS were performed as stated above.

### 2.4. TAM Experiments

The ligand-structure drawing and aligning were carried out with BIOVIA Discovery Studio Visualizer, a molecular modeling software package available from Dassault Systemes BIOVIA Software Inc (San Diego, CA, USA) [26]. The file of the JDTic-bound KOR crystal structure, 4djh, was downloaded from PDB (http://www.rcsb.org) (accessed on 20 April 2020). For docking of **6**, the PDB file of 4djh was opened at the interface of BIOVIA Discovery Studio Visualizer. By unchecking “Side Chain” at “Protein Groups”, all of the side chains of the receptor protein were hidden from view, and only the backbone was displayed for clarity. The template was manually docked onto the binding site of the KOR, where the morphinan core of the template was superimposed to the tetrahydroisoquinoline (Tiq) moiety of JDTic, a pre-positioned reference ligand. The aromatic rings of the morphinan core matched with the Tiq moiety, and the 4-(2-hydroxyphenyl)piperidinyl moiety positioned above the template (Figure S1).

### 2.5. Plasma Stability Tests

Plasma stabilities were tested in 50% and/or 95% of human plasma obtained from healthy donors. Analogs **5** and **6** were dissolved in a 10% methanol solution to a final concentration of 0.39 μM and 0.57 μM, respectively. As an internal standard, Z-Lys-OH was added to the stock solution. For the tests, 50 μL of **5**, 50 μL of **6**, and 5 μL of **6** were added to 50 μL, 50 μL, and 95 μL of human plasma, respectively. To validate the plasma activity (positive control), 50 μL of an EM-1 solution (0.78 μM) was added to the first test solution containing **5** in 50% human plasma. Test solutions were incubated at 37 ± 1 °C. After incubation for durations of 1, 2 and 6 h, 1, 2, 3, 4, 7, 21, 42 d, 200 uL of ethanol was added to quench the reaction. The turbid solution was cooled at 4 °C for 10 min and was centrifugated at 2000× *g* for 10 min. The supernatant (15 μL) was analyzed by LC/MS (LCMS-2010EV Shimadzu system) using a C-12 column (Phenomenex Jupiter 4 μ Proteo 90 A, 25 cm × 2 mm, 4 μm) at 37 °C. Gradient systems analyzing **5** and **6** were 3–75% of solution B (0.05% HCOOH in acetonitrile) within 48 min and 75–97% within an additional 9 min, and 3–51% of solution B within 40 min and 51–97% within an additional 20 min, respectively, at a flow rate of 0.3 mL/min. Absorbance was detected at 210 nm and positive electron spray (ESI^+^) was used for the ionization. The peak areas and masses of the test compounds and Z-Lys-OH (internal standard) were determined by LC/MS. The tests were performed more than two times to confirm the reproducibility of obtained results. Overall long-term stabilities were plotted by the peptide concentration (%) calculated from the ratio of two peak areas of **5** (or **6**) and Z-Lys-OH referenced to the value at 0 min.

## 3. Results and Discussion

### 3.1. Synthesis

Enkephalin analogs were prepared by standard liquid phase peptide synthesis using N^α^-Boc-chemistry with high crude purity and isolated by preparative reversed phase–high-performance liquid chromatography (RP-HPLC) using a C-18 column to afford > 97% purity, as described in earlier publications including the data for the MOR and DOR except **MR119** (**7**, molecular formula C_42_H_55_BrN_6_O_6_; (M-TFA + H)^+^ obsd 821.3415, cald 819.3445; HPLC *t*_R_ 25.4 min, aLOGPs 4.25) [14,15,22]. Their structures were validated by HRMS and NMR spectroscopy in the publications. Synthesized analogs were evaluated for their binding affinities and functional activities at the KOR by competition binding assays and [^35^S]GTPγS assays, respectively.

### 3.2. Binding Affinities

Assay results showed that modifications of **LYS729** (**1**), an enkephalin-like tetrapeptide amide, at positions 1, 2 and 4 with Dmt, DNle and Phe(*p*-X) increased the binding affinities at the KOR in all analogs, while C-terminal modification with a Ppp group in **LYS436** (**2**) did not affect affinities (Table 1). The increase of binding affinities occurred also at the MOR and DOR, as published earlier [14]. It is clear that having a Dmt residue at position 1 increased affinities for the KOR in their ligands by comparison of affinities of **2** (*K*_i_ = 220 nM) and **LYS540** (**3**) (*K*_i_ = 21 nM). It is also clear that substitution of position 2 with a DNle, a Met isosteric non-natural amino acid, contributed to the interaction at the KOR in **LYS644** (**4**) (*K*_i_ = 4.8 nM) and **1** (*K*_i_ = 1.3 nM). Analogs **3** (*K*_i_ = 21 nM), **8** (*K*_i_ = 2.4 nM), and **LYS745** (**9**) (*K*_i_ = 3.8 nM), which have DAla or DTic at postion 2 showed lower binding affinities than their counterpart analogs with DNle, **6** and **4**.

Substitution of the para position of the phenyl ring of Phe residue at position 4 with a fluorine or a chlorine increased binding affinities in all cases, especially in **5** (*K*_i_ = 0.89 nM), whereas a small decrease was observed in the brominated ligand, **7** (*K*_i_ = 7.4 nM). Among these ligands, **5** and **6**, where three effective substituents, Dmt, DNle, and Phe(*p*-F or *p*-Cl), located at positions 1, 2, and 4, respectively, were lead ligands with good binding affinities at the KOR, as well as the MOR (*K*_i_ = 0.02 nM and 0.10 nM, respectively) and the DOR (*K*_i_ = 0.40 nM and 0.08 nM, respectively) [14,15].

### 3.3. Functional Activity: [^35^S]GTPγS Assay

Analogs **1**–**6** were tested for their functional activities at the KOR in the [^35^S]GTPγS assay (Table 2). As expected, **1** and **2**, with lower affinities, did not show significant stimulation (E_max_ < 30%) or inhibition (I_max_ < 10%) at a high concentration (10 µM) compared to the vehicle. The functional assay results were well correlated with the binding affinities. It was also confirmed that respective substitutions of positions 1, 2 and 4 with Dmt, DNle and Phe(*p*-X) increased functional activities as well as binding affinities. More importantly, the substitutions resulted in the reversal of functional activity from a mixed partial agonist/antagonist (**3**–**5)** to a full antagonist (**6**) for the KOR. Although there were increases of functional activities in **4** (EC_50_ = 210 nM, E_max_ = 41%; IC_50_ = 386 nM, I_max_ = 57%) with the DNle substitution and **5** (EC_50_ = 21,1 nM, E_max_ = 39%; IC_50_ = 59.8 nM, I_max_ = 65%) with the DNle and Phe(*p*-F) substitutions, their biological profiles as a mixed agonist/antagonist of **3** (EC_50_ = 538 nM, E_max_ = 39%; IC_50_ = 521 nM, I_max_ = 64%) were conserved in these analogs.

**Figure 2:**
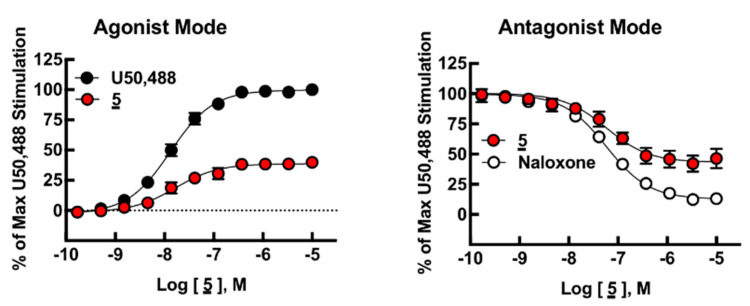
[^35^S]GTPγS assays in hKOR-CHO cells. Agonist mode dose–response curves of **5** and U50,488 (**left**). Antagonist mode dose–response curves of **5** and naloxone (**right**). Data points represent mean ± SEM of the percent of U50,488 stimulation. From n = 3 independent experiments for both modes. Three-variable non-linear regression curves fit by GraphPad Prism.

As described, **5** is a highly potent multifunctional ligand showing a unique biological profile as a MOR/DOR agonist and KOR partial agonist/antagonist (Figure 2). Interestingly, **6**, which has a Phe(*p*-F) residue replaced with a Phe(*p*-Cl) residue, did not show agonist activity (E_max_ < 10% at 10 μM), but rather full antagonist activity, with a higher I_max_ (122%) than naloxone at the receptor. Therefore, **6** was the only ligand to exhibit full antagonist activity at the KOR. Another functional assay using high-throughput TANGO technology showed moderate agonist activities for analogs **8** and **9** (EC_50_ = 130 nM, and 230 nM, respectively) (Table S1) [27,28]. It was also shown that **7** with the substitution of a Phe(*p*-Br) residue reduced its agonist activity to the micromolar range (EC_50_ = 4.6 µM).

Although all analogs were designed based on the structure of endogenous enkephalin for the MOR and DOR, it seems to be necessary to ensure the target receptor specificity because of distinct modifications at multiple positions. Before this SAR study for the KOR, a lead ligand **5** was screened at 43 receptors including three subtypes of opioid receptors, 5HT receptors, etc., to confirm its specificity for the MOR and DOR [27,29]. While the screen was limited to 43 receptors, the blinded analysis showed that **5** interacts with all three subtypes of opioid receptors, including the KOR, but not with any other 38 off-target receptors except the D3 (*K*_i_ = 980 nM) and NET transporter (*K*_i_ = 3400 nM) (Supporting Information Table S2). This off-target receptor screening was the starting point of this study on multifunctional activity of enkephalin analogs.

Considering the well-known SAR of enkephalin and dynorphin analogs, the sub-nanomolar binding affinity and partial agonist/antagonist activity of **5** for the KOR was a surprising result. It was also found that **6**, with a Phe(*p*-Cl) at position 4, could reverse the partial agonist activity to a pure antagonist activity at the KOR with retained binding affinity. This was another surprising SAR result, demonstrating halogen effects on KOR interactions [30,31,32]. On the basis of the SAR result, it is possible that halogen atoms influence the nature of the aromatic ring of the Phe residue and the peptide’s conformation due to differing electronegativity and steric effects (carbon-halogen bond lengths: F = 1.40, Cl = 1.79, Br = 1.97, I = 2.16 Å) together with the overall changes in the lipophilicity. An example is that a fluorinated-fentanyl analog worked better under pathophysiological conditions of injury or inflammation, i.e., under acidic conditions, than under healthy conditions due to a strong negative charge [33].

### 3.4. Modeling Experiments: TAM Approach

To obtain a better understanding of the structural basis for the unique KOR antagonism of **6**, modeling experiments using the TAM approach were performed. TAM is a newly published ligand-based modeling approach for structural correlations as well as SAR analysis of opioid ligands [21,34]. As an innovative approach, TAM emphasizes the important role of the backbone or the scaffold of a ligand for ligand–receptor interactions, which differs greatly from conventional modeling methods, which mostly focus on the side chains or the functional groups. As discussed in previous publications, TAM can effectively assist the SAR interpretations of a wide variety of opioid ligands [21].

The proper alignment of a ligand with a relevant template is a critical process of TAM. Here, we apply the previously constructed MOR-agonist template for the alignment of **6**. Because of the presence of delta- and kappa-specific areas within the scaffold, the MOR-agonist template can be applied for the alignment of DOR and KOR ligands as well. For information about the kappa-specific area, as well as examples of the related alignments, see references [21,35].

**Figure 3:**
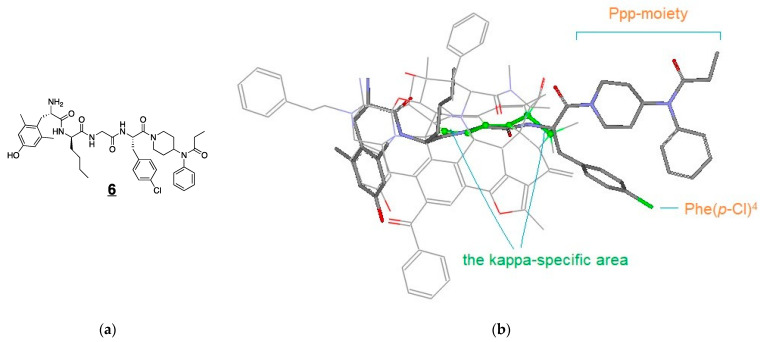
The structure of **6** (**a**) and alignment of the backbones of **6** along the kappa-specific area (light green) of the MOR agonist template (**b**).

Thus, we took a two-step process in investigating the unique KOR antagonism of **6** on the roles of: (1) the Ppp moiety at the C-terminus and (2) the halogen atom of the Phe residue at position 4. Initially, we aligned the backbones of DNle^2^ through Phe(*p*-Cl)^4^ of **6** along the kappa-specific area of the MOR-agonist template (Figure 3). In this alignment, the side chain of DNle^2^ takes a special position as defined by many other KOR ligands, including the bound ligand of the crystal structure of a KOR complex (unpublished results), which may account for the enhanced KOR-binding of DNle^2^. Upon alignment, the *p*-chlorophenyl group of **6** is positioned below the kappa-specific area, while the bulky and rigid Ppp moiety extends beyond the main scaffold. From this alignment, the conformation of **6** relative to the template is revealed so as to facilitate the further modeling process (Figure 3).

**Figure 4:**
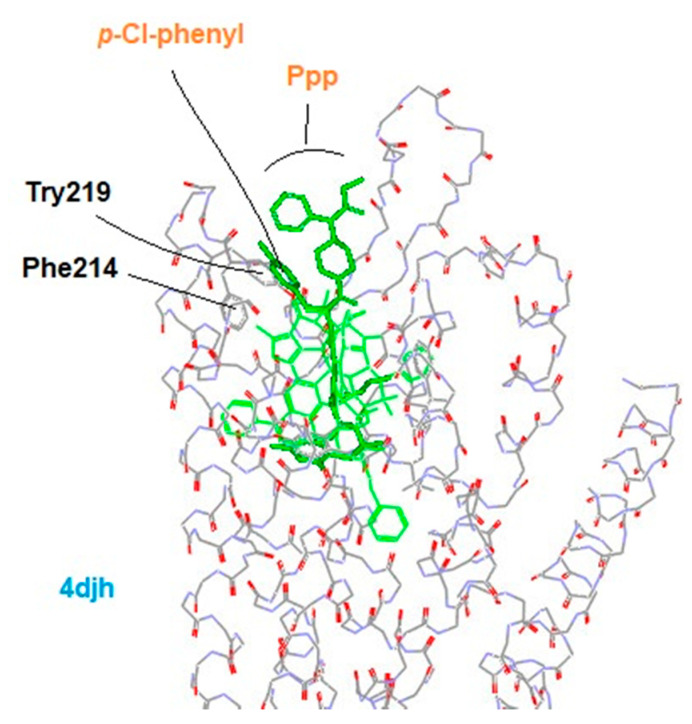
Docking of **6** as aligned with the template (light green) onto the binding pocket of a KOR-JDTic complex (4djh).

The structure of **6** was then aligned with the template and docked onto the binding pocket of the crystal structure of a KOR-JDTic complex (4djh) manually, where the template’s morphinan core was superimposed to the Tic moiety of JDTic (Figure 4 and Supplementary Materials Figure S1). Please note that the template here is used as a reference for **6** to be adequately positioned, from which the binding pose of this ligand at the binding site is inferred. For further information regarding the docking process of TAM, see reference [21]. On the basis this docking, the approximate positions of *p*-chlorophenyl and the Ppp moieties of **6** become apparent. As we can see in Figure 4, the *p*-chlorophenyl moiety is located near Tyr^219^ and Phe^214^ of the KOR structure, while the Ppp moiety is close to the opening of the binding pocket.

**Figure 5:**
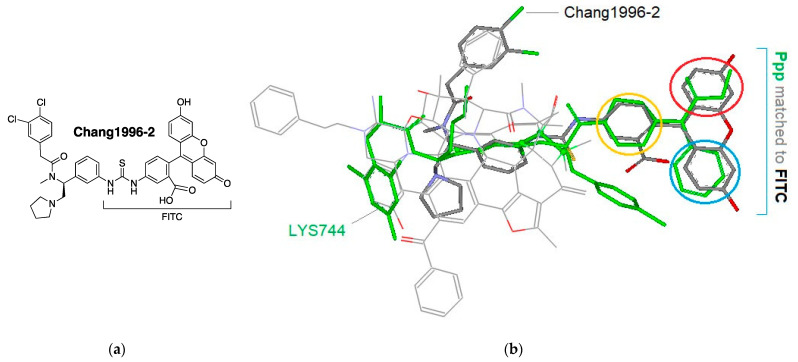
Structural correlation between **6** (**LYS744**) and Chang1996-2. The structure of Chang1996-2 (**a**) and co-alignment of Chang1996-2 (grey) and **6** (light green) with the template (**b**).

In the TAM process, we often apply special ligands as additional references in addition to using a template to assist with the structural correlation and analysis. To understand the role of the Ppp moiety, we selected Chang1996-2 as a reference. Chang1996-2 belongs to a group of arylacetamide-derived fluorescent probes with pure KOR agonist activity, the structures of which are composed of two major parts: a potent KOR agonist motif along with a bulky and rigid fluorescein isothiocyanate (FITC) moiety (Figure 5) [36]. After Chang1996-2 and **6** were co-aligned with the template, the close structural correlation between the FITC and the Ppp moieties became apparent, where the piperidine ring, phenyl, and propyl amide groups of Ppp are well matched with the three aromatic rings of FITC, respectively (circled in yellow, blue and red in Figure 5). This comparison suggests that the Ppp moiety is responsible for the KOR binding of the Ppp-attached peptides, as it can take the same binding pose and interacts with the binding site similarly as the FITC moiety. On the other hand, the Ppp moiety does not appear to be the direct cause for the KOR antagonism of the Ppp-attached peptides. As mentioned earlier, Chang1996-2 acts as a KOR agonist, which suggests that its FITC moiety is KOR agonistic in nature. Accordingly, the closely correlated structure of the Ppp moiety is considered to be agonistic in nature as well. This speculation can also be supported with the fact that the KOR antagonism of the Ppp-attached peptides is largely dependent on the substituents of Phe^4^ (Table 2).

Since the Ppp moiety does not appear to be responsible for the unique antagonism at the KOR, our query was now narrowed to the Phe(*p*-X) residue at the 4th position. F-substitution is well known for its strong electron-withdrawing effect on aromatic rings, which may directly or indirectly affect the ligand–receptor interactions. However, the overall effects did not seem to be purely electrostatic in this case, as halogen substitution on the KOR antagonism showed the tendency of Cl > F > H (Table 2). The Phe(*p*-F)^4^ substituted analog **5** displayed a mixed partial antagonism (I_max_ = 65%)/agonism (E_max_ = 39%), while the Phe(*p*-Cl)^4^ substituted analog **6** acted as a full antagonist (I_max_ = 122%) with no agonist activity (E_max_ < 10%) for the KOR. Based on this substituent-dependent functional transformation, we then hypothesized: (1) there are two distinct conformations of the aromatic ring of Phe^4^ at the binding site: one responsible for the KOR agonism, and the other for the antagonism; (2) the two conformational states exist in a dynamic equilibrium, depending on the substituent effect from Phe^4^. For instance, the Phe(*p*-F)^4^ of **5** with the mixed functions can present the two conformations simultaneously. For **6**, however, the balance is largely shifted to the antagonistic side due to the different substitution. Considering the close proximity of Phe^4^ to Tyr^219^ and Phe^214^ at the binding site, their aromatic rings can undergo aromatic pi-stacking interactions, promoting the antagonistic conformation (Figure 4) [37,38]. In addition, due to the attenuated electron-negativity as well as the potential lone-pair conjugation of a chlorine atom, the *p*-Cl-substitution presumably enhances the pi-stacking, and thus the antagonistic conformation. By this mechanism, the significant KOR antagonism of **6**, as compared to **5**, is accounted for.

Additionally, this pi-stacking interaction hypothesis can also be supported with the literature-reported SAR data. The indole ring of a Trp residue is a large pi-conjugated system, which would presumably favor the pi-stacking interactions. Indeed, this prediction is evidenced by several of the Trp^4^-substituted dynorphin-A analogs, where Trp^4^ substitution is shown to give rise to significant KOR antagonism of the related analogs [39,40,41].

### 3.5. In Vitro Plasma Stability

**Figure 6:**
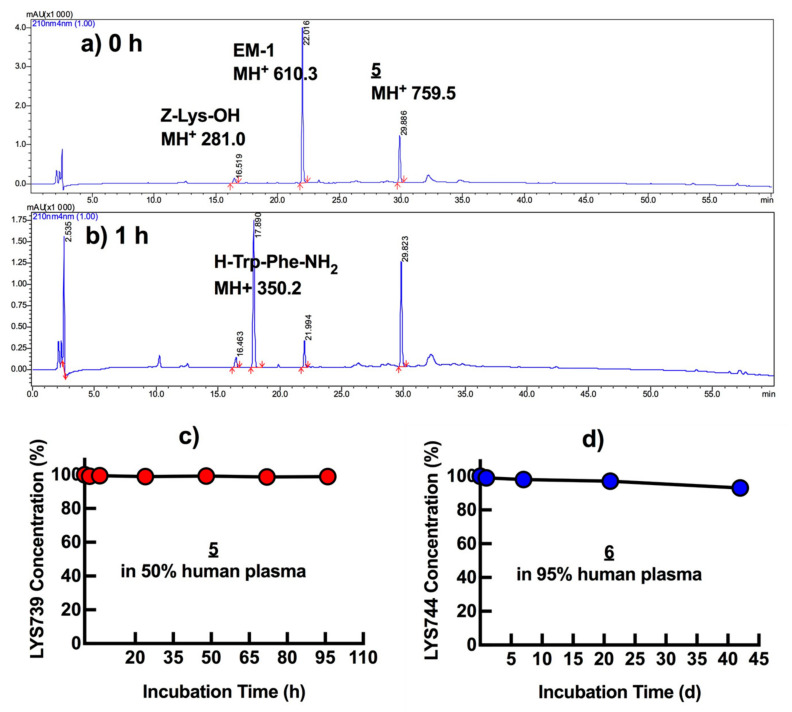
Stabilities of **5** and **6** in human plasma at 37 °C. (**a**) HPLC profile of **5**, EM-1, and Z-Lys-OH (internal standard); (**b**) after 1 h incubation at 37 °C. Gradient: 3–75% of B solution (0.05% HCOOH in acetonitrile) in A solution (0.05% HCOOH in water) within 48 min; (**c**) overall long-term stabilities of **5** in 50% human plasma; and (**d**) **6** in 95% human plasma.

This series of analogs possess high potential for increased bioavailability due to their lipophilic character, non-natural amino acid substitutions, and C-terminal modification. Although plasma stability does not fully represent metabolic stability, plasma is one of two major sites where most peptides and proteins are metabolized, and prolongation of half-life is often a prerequisite for drug candidates [42]. Additionally, it is important to ensure that BBB permeability and in vivo studies using matrix, i.e., plasma, will not be misled due to potential degradation and/or protein binding issues [43]. Therefore, our lead ligands **5** and **6** were first tested for their stabilities in 50% and/or 95% human plasma. In these tests, both analogs showed very high plasma stabilities (Figure 6). There was no evident degradation detected on LC/MS analyses after long-term incubation (**6** concentration > 90% after 42 days in 95% human plasma) (Figures S2–S4). Analog **5** retained high concentration (>98%, MH^+^ 759.5) after 4-day incubation, while EM-1 (H-Tyr-Pro-Trp-Phe-NH_2_, MH^+^ 610.3), a MOR agonist tetrapeptide with a poor metabolic stability, degraded into H-Trp-Phe-NH_2_ (MH^+^ 350.2) within 1 h [44]. The degradation of EM-1 also confirmed the activity of plasma we used.

The high plasma stabilities of these two analogs represent promising results as compounds that degrade rapidly possess poor in vivo efficacy. In fact, high analgesic efficacy of these new chemical entities was shown in our previous in vivo tests [14]. Analog **8** showed high anti-hyperalgesic and anti-allodynic effects in neuropathic pain animal models with spinal nerve ligation (SNL) surgery in a dose-dependent manner [14]. Analog **5** also showed enhanced neuroprotection in a mouse middle cerebral artery occlusion (MCAO) stroke model by decreasing brain infarct and edema ratios [45]. Based on these results, this series of enkephalin analogs was considered to have good antinociceptive effects coupled with a potential neuroprotective effect.

Analog **6** was evaluated for BBB permeability in an in vitro BBB permeability assay using a Madin-Darby canine kidney (MDCK)-MDR1 cell monolayer that was modified to overexpress P-glycoprotein [46]. In the assay, apparent permeability (P_app_) across the cell monolayer in both directions, apical-to-basolateral and basolateral-to-apical, was 0.38 and 0.83 × 10^−6^ cm/s, respectively, with a high efflux ratio (>2.17). There were insufficient recoveries (<50%) observed, which might be caused by non-specific binding, cellular retention, or other issues. Nonetheless the high plasma stability, the low P_app_ and high efflux ratio indicate that **6** possesses low potential to penetrate the BBB, and the efflux may impede the penetration. Currently, various in vivo tests for **6** are in progress to verify the benefits of blocking the KOR while retaining MOR agonist activity.

## 4. Conclusions

While developing highly lipophilic and potent MOR/DOR agonists with improved plasma stability, we were able to discover a new biological profile of ligand **6**, as a MOR/DOR agonist and KOR antagonist. The unexpected KOR antagonist activity of **6** shows that it is possible to discover a highly integrated peptide molecule for opioid receptors by simple modifications of a short peptide scaffold like enkephalin, an endogenous ligand for the DOR. The simple modifications, namely attachment of a Ppp moiety to the C-terminus along with halogen substitution on the aromatic ring of Phe^4^, also resulted in a large increase of lipophilicity and plasma stability. TAM and docking experiments suggested that a key structural feature for the blockade of the KOR might be a halogen substitution, specifically Cl, on the phenyl ring of Phe^4^ in the presence of a Ppp moiety at the C-terminus. Taken together, the Ppp moiety is suggested as a good tool to optimize activities and physicochemical properties simultaneously by being conjugated to a peptide scaffold via a standard peptide coupling procedure. Our lead ligand **6** is a compound that has therapeutic potential for the treatment of pathological KOR activation in chronic pain states by modulating KOR-induced side effects as a full antagonist while retaining analgesic efficacy attributed to its MOR/DOR agonist activity.

## Figures and Tables

**Table 1:** Structures and binding affinities of enkephalin analogs **1**–**9** at the KOR, MOR, and DOR.

Analog	Structure	hKOR ^1^		rMOR ^5^	hDOR ^5^
		[^3^H]U69,593 ^2^		[^3^H]DAMGO	[^3^H]DPDPE
		*K*_i_ (nM) ^3^	−logIC_50_^4^	*K*_i_ (nM)	*K*_i_ (nM)
**LYS729 (1)**	Tyr-DAla-Gly-Phe-NH_2_	230	6.65 ± 0.13	2.8	300
**LYS436** (**2**)	Tyr-DAla-Gly-Phe-Ppp	220	6.66 ± 0.10	23	0.69
**LYS540** (**3**)	Dmt-DAla-Gly-Phe-Ppp	21	7.68 ± 0.24	0.38	0.36
**LYS644** (**4**)	Dmt-DNle-Gly-Phe-Ppp	4.8	8.32 ± 0.12	0.39	0.18
**LYS739** (**5**)	Dmt-DNle-Gly-Phe(*p*-F)-Ppp	0.89	10.5 ± 0.10	0.02	0.40
**LYS744** (**6**)	Dmt-DNle-Gly-Phe(*p*-Cl)-Ppp	1.3	8.89 ± 0.15	0.10	0.08
**MR119** (**7**)	Dmt-DNle-Gly-Phe(*p*-Br)-Ppp	7.4	8.13 ± 0.12	1.5	1.1
**LYS707** (**8**)	Dmt-DAla-Gly-Phe(*p*-Cl)-Ppp	2.4	8.62 ± 0.07	0.14	0.14
**LYS745** (**9**)	Dmt-DTic-Gly-Phe(*p*-Cl)-Ppp	3.8	8.42 ± 0.05	0.15	0.11
	Salvinorin A	2.4	8.62 ± 0.10	-	-

^1^ Radioligand competitive binding assays were performed in membranes from stable HEK cells that constitutively expressed the KOR. ^2^ *K*_d_ = 1.07 ± 0.10 nM. ^3^ Calculated by the Cheng-Prusoff equation. ^4^ Data are presented as the means ± SEM from n = 3 independent experiments using a non-linear regression analysis (GraphPad Prism). ^5^ Data except **7** are from [14,15] to be shown for comparison. Analog **7** was synthesized later and tested for binding affinities at the receptors in the assays as described in the references.

**Table 2:** [^35^S]GTPγS assays of enkephalin analogs at the KOR.

Analog	KOR ^1^			
	Agonist Mode		Antagonist Mode ^2^	
	EC_50_ (nM) ^3^	E_max_ (%) ^4^	IC_50_ (nM) ^5^	I_max_ (%) ^6^
**1**	-	<30 ^7^	-	<10 ^8^
**2**	-	<10 ^7^	-	<10 ^8^
**3**	538 ± 75	39	521 ± 71	64
**4**	210 ± 51	41	386 ± 35	57
**5**	21.1 ± 7.2	39	59.8 ± 10.0	65
**6**	-	<10 ^7^	52.4 ± 1.2	122
naloxone	-	-	58.6 ± 4.4	100
U50,488	13.8 ± 2.5	100	-	-

^1^ Expressed in hKOR-CHO cells. ^2^ Measures the analogs’ ability to block stimulation by 100 nM U50,488. ^3^ The concentration required to afford 50% stimulation. ^4^ The percentage relative to the stimulation of 1 μM U50,488. ^5^ The concentration required to afford 50% inhibition. ^6^ The percentage relative to the inhibition of 10 μM naloxone. ^7^ Percent stimulation at 10 μM. ^8^ Percent inhibition at 10 μM. Values normalized to the maximum stimulation caused by U50,488. Data reported as the mean ± SEM from n = 3 independent experiments for both modes.

## Data Availability

Data is contained within the article or supplementary material.

## Supplementary Materials

The following are available online at https://www.mdpi.com/article/10.3390/biomedicines9060625/s1, Table S1: Agonist activities of analogs in TANGO assay; Table S2: Binding affinities (*K*_i_, nM) of **LYS739** (**5**) at 40 off-target receptors; Figure S1: Docking of **LYS744** (**6**) as aligned with the template onto the binding site of KOR (4djh) (green, left); Figure S2: Stability of **LYS739** (**5**) in 50% Human Plasma; Figure S3: Stability of **LYS744** (**6**) in 50% Human Plasma; S4: Stability of **LYS744** (**6**) in 95% Human Plasma.
