# Supplementary material for: Multifunctional Enkephalin Analogs with a New Biological Profile: MOR/DOR Agonism and KOR Antagonism
Source: Biomedicines. 2021 May 31;9(6):625. doi: 10.3390/biomedicines9060625 (PMC8229567; doi:10.3390/biomedicines9060625)
Supplement: Supplementary file 1 [file biomedicines-09-00625-s001.zip › biomedicines-1225298-SI.pdf]

Multifunctional enkephalin analogs with a new biological profile: MOR/DOR agonism and KOR antagonism

Yeon Sun Lee [1,*], Michael Remesic [2], Cyf Ramos-Colon [2], Zhijun Wu [3], Justin LaVigne [1], Gabriella Molnar [1], Dagmara Tymecka [4], Aleksandra Misicka [4], John M. Streicher [1], Victor J. Hruby [2], and Frank Porreca [1]

[1] Department of Pharmacology, University of Arizona, Tucson, AZ 85724, USA
[2] Department of Chemistry and Biochemistry, University of Arizona, Tucson, AZ 85721, USA
[3] ABC Resource, Plainsboro, NJ 08536, USA
[4] Faculty of Chemistry, University of Warsaw, Pasteura, PL-02-093 Warsaw, Poland
* Correspondence: yeon@email.arizona.edu; Tel: +1-520-626-2820

Table S1. Agonist activities of analogues in TANGO assay*

Analog	TANGO, Log EC_{50}	EC_{50}
**LYS729** (1)	-5.23 ± 0.40	5,900
**MR119** (7)	-5.34 ± 0.25	4,600
**LYS707** (8)	-6.88 ± 0.20	130
**LYS745** (9)	-6.64 ± 0.14	230
Salvinorin	-7.55	28

* Data was generously provided by the National Institute of Mental Health's Psychoactive Drug Screening Program, Contract # HHSN-271-2018-00023-C (NIMH PDSP). The NIMH PDSP is Directed by Bryan L. Roth MD, PhD at the University of North Carolina at Chapel Hill and Project Officer Jamie Driscoll at NIMH, Bethesda MD, USA.
For experimental details please refer to the PDSP web site https://pdsp.unc.edu/pdspweb/.

Table S2. Binding affinities (K_i, nM) of **LYS739** (5) at 40 off-target receptors

5-HT1A	5-HT1B	5-HT1D	5-HT1E	5-HT2A	5-HT2B	5-HT2C	5-HT3	5-HT5A	5-HT6	5-HT7
n.c.	n.c.	n.c.	n.c.	n.c.	n.c.	n.c.	n.c.	n.c.	n.c.	n.c.

D1	D2	D3	D4	D5	SERT	NET	DAT	GABA	H1	H2
n.c.	n.c.	980	> 10,000	n.c.	n.c.	3,400	n.c.	n.c.	n.c.	n.c.

H3	Alpha1A	Alpha1B	Alpha1D	Alpha2A	Alpha2B	Alpha2C	Beta1	Beta2	M1	M2
n.c.	n.c.	n.c.	n.c.	n.c.	n.c.	n.c.	n.c.	n.c.	n.c.	n.c.

M3	M4	M5	Sigma1	Sigma2	Beta3	BZP Rat Brain Site	PBR
n.c.	n.c.	n.c.	n.c.	n.c.	n.c.	n.c.	n.c.

n.c.; no competition (<50%) at 10 uM. n = 4 determinations for compound tested at receptor. Significant inhibition is considered > 50%. In cases where negative inhibition (-) is seen, this represents a stimulation of binding. Occasionally, compounds at high concentrations will nonspecifically increase binding. Data represent K_i (nM) values obtained from non-linear regression of radioligand competition binding isotherms. K_i values are calculated from best fit IC_{50} values using the Cheng-Prusoff equation.

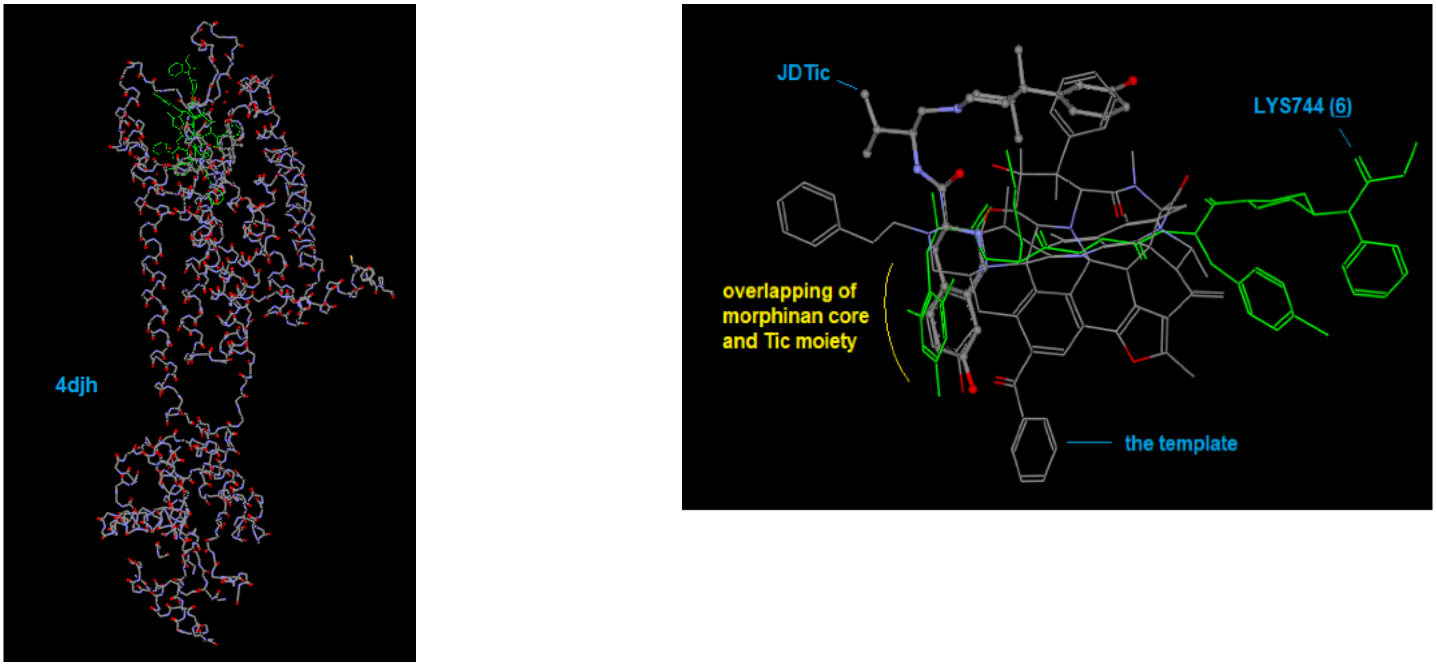

Figure S1. Docking of **LYS744** (**6**) as aligned with the template onto the binding site of KOR (4djh) (green, left). The morphinan core of template is superimposed to the Tiq moiety of JDTic (right).

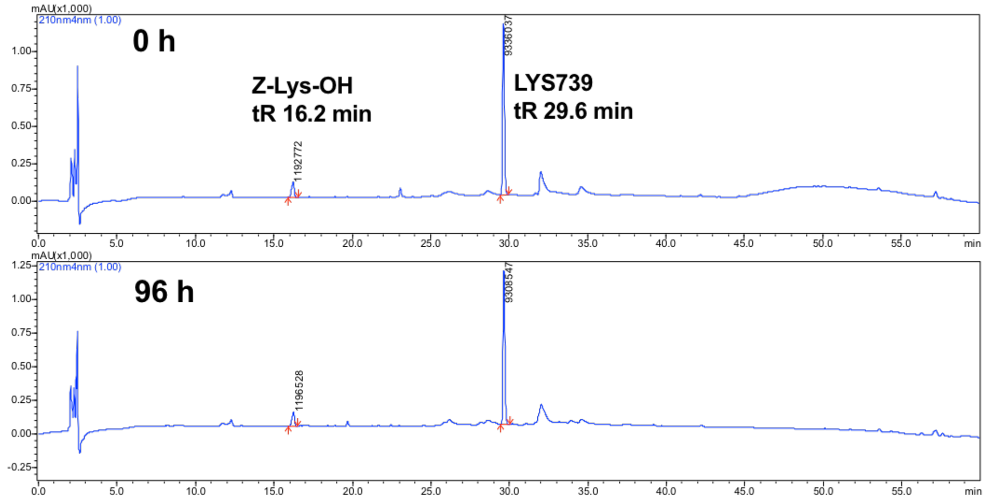

MS of the peak at 29.6 min (below)

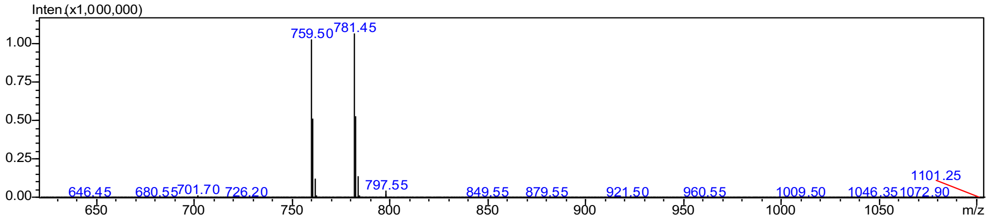

MS of the peak at 16.2 min (below)

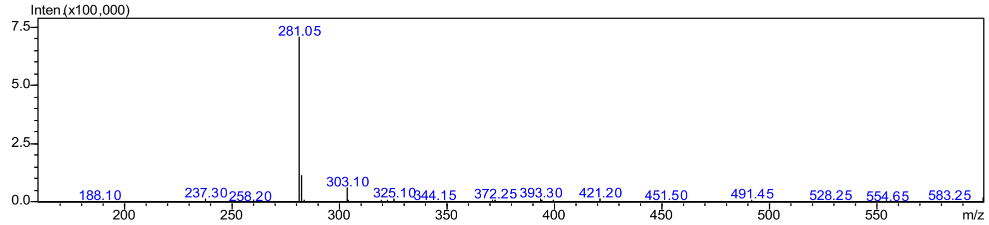

Figure S2. Stability of **LYS739** (**5**) in 50% Human Plasma. The HPLC profile after 96 h-incubation at 37 ºC showed no degradation. Gradient: 3-75% B in 48 min and -95% in 59 min, A: water solution containing 0.05% HCOOH, B: acetonitrile solution containing 0.05% HCOOH.

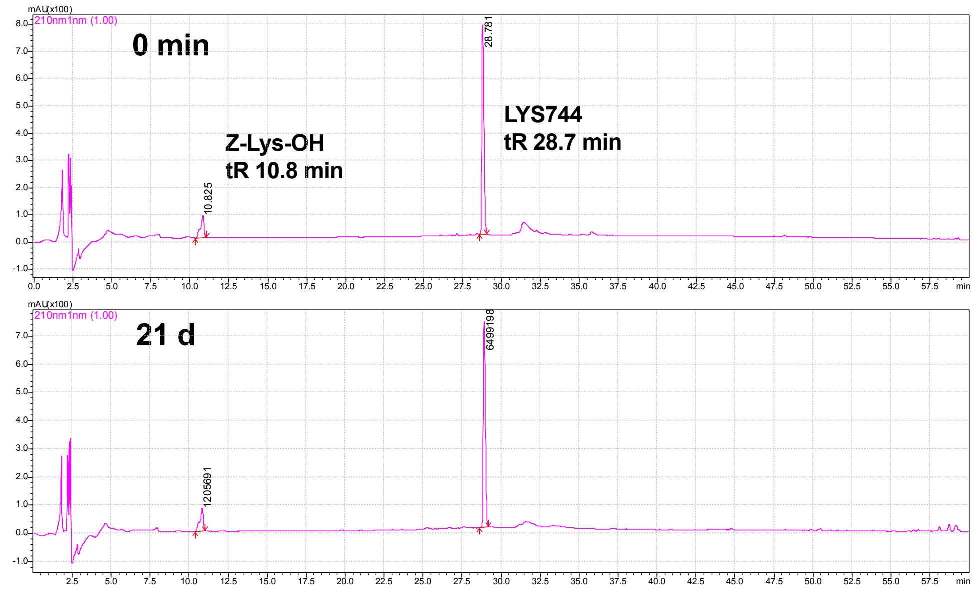

MS of peak at 28.7 min; below (positive and negative, respectively)

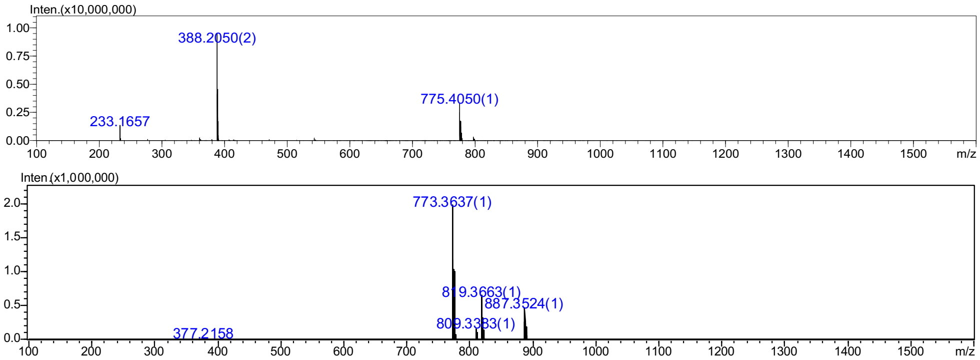

MS of peak at 10.8 min; below (positive and negative, respectively)

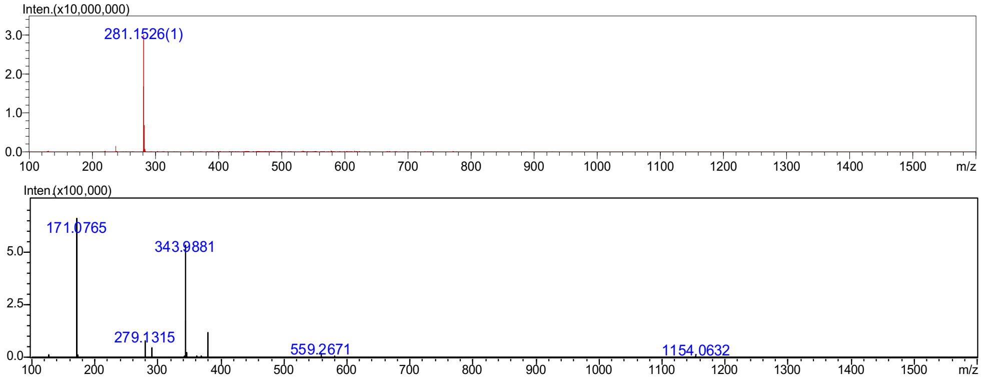

Figure S3. Stability of **LYS744** (<u>**6**</u>) in 50% Human Plasma. The HPLC profile after 21 d-incubation at 37 ºC showed no degradation. Gradient: 3-51% B in 51 min and -97% in 59 min, A: water containing 0.05% HCOOH, B: acetonitrile containing 0.05% HCOOH.

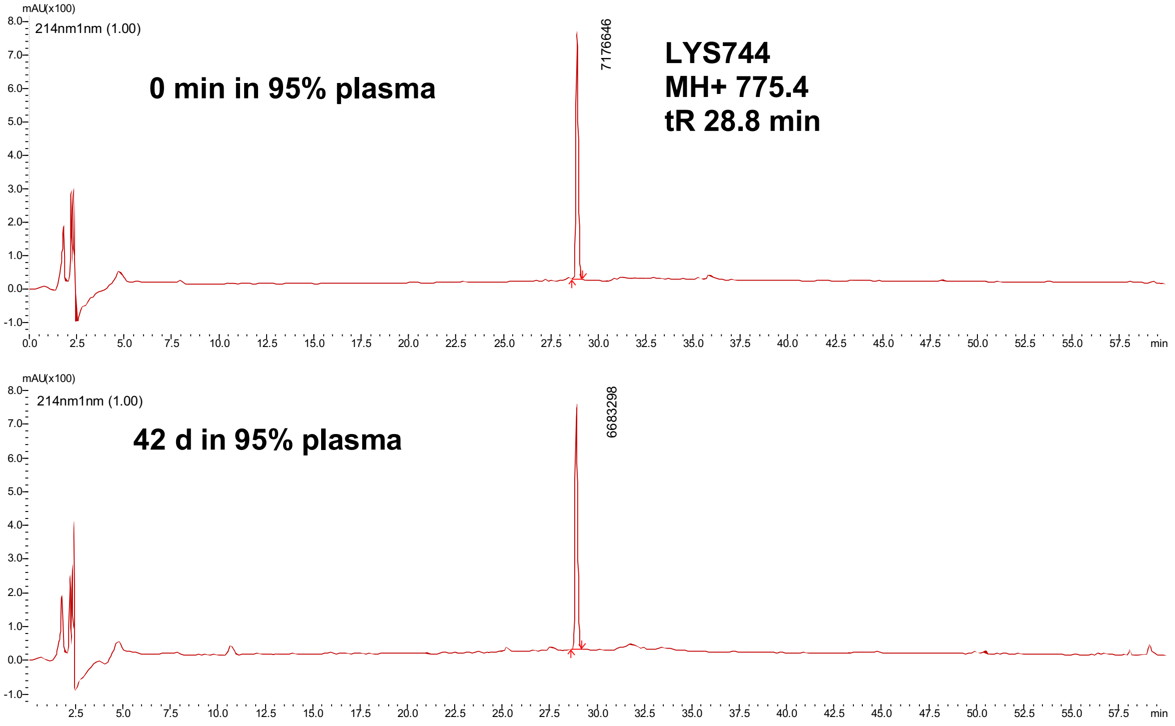

Figure S4. Stability of **LYS744** (**6**) in 95% Human Plasma. The HPLC profile after 42 d-incubation at 37 °C showed no degradation. Gradient: 3-51% B in 40 min and -97% in 60 min, A: water containing 0.05% HCOOH, B: acetonitrile containing 0.05% HCOOH.
